# Safety and tolerability of nintedanib in patients with systemic sclerosis-associated interstitial lung disease: data from the SENSCIS trial

**DOI:** 10.1136/annrheumdis-2020-217331

**Published:** 2020-08-05

**Authors:** James R Seibold, Toby M Maher, Kristin B Highland, Shervin Assassi, Arata Azuma, Laura Kathleen Hummers, Ulrich Costabel, Ute von Wangenheim, Veronika Kohlbrenner, Martina Gahlemann, Margarida Alves, Oliver Distler, the SENSCIS trial investigators

**Affiliations:** 1 Scleroderma Research Consultants, LLC, Aiken, South Carolina, USA; 2 National Heart and Lung Institute, Imperial College London, London, UK; 3 National Institute for Health Research Clinical Research Facility, Royal Brompton Hospital, London, UK; 4 Keck School of Medicine, University of Southern California, Los Angeles, California, USA; 5 Respiratory Institute, Cleveland Clinic, Cleveland, Ohio, USA; 6 University of Texas Houston Medical School, Houston, Texas, USA; 7 Department of Pulmonary Medicine and Oncology, Graduate School of Medicine, Nippon Medical School, Tokyo, Japan; 8 Division of Rheumatology, Johns Hopkins University School of Medicine, Baltimore, Maryland, USA; 9 Interstitial and Rare Lung Disease Unit, Department of Pneumology, Ruhrlandklinik, University Hospital Essen, Essen, Germany; 10 Boehringer Ingelheim Pharma GmbH & Co. KG, Biberach, Germany; 11 Boehringer Ingelheim Pharmaceuticals, Inc, Ridgefield, Connecticut, USA; 12 Boehringer Ingelheim (Schweiz) GmbH, Basel, Switzerland; 13 Boehringer Ingelheim International GmbH, Ingelheim am Rhein, Germany; 14 Department of Rheumatology, University Hospital Zurich, Zurich, Switzerland

**Keywords:** autoimmune diseases, systemic sclerosis, pulmonary fibrosis

## Abstract

**Objectives:**

To characterise the safety and tolerability of nintedanib and the dose adjustments used to manage adverse events in patients with systemic sclerosis-associated interstitial lung disease (SSc-ILD).

**Methods:**

In the SENSCIS trial, patients with SSc-ILD were randomised to receive nintedanib 150 mg two times per day or placebo. To manage adverse events, treatment could be interrupted or the dose reduced to 100 mg two times per day. We assessed adverse events and dose adjustments over 52 weeks.

**Results:**

A total of 576 patients received nintedanib (n=288) or placebo (n=288). The most common adverse event was diarrhoea, reported in 75.7% of patients in the nintedanib group and 31.6% in the placebo group; diarrhoea led to permanent treatment discontinuation in 6.9% and 0.3% of patients in the nintedanib and placebo groups, respectively. In the nintedanib and placebo groups, respectively, 48.3% and 12.2% of patients had ≥1 dose reduction and/or treatment interruption, and adverse events led to permanent discontinuation of the trial drug in 16.0% and 8.7% of patients. The adverse events associated with nintedanib were similar across subgroups defined by age, sex, race and weight. The rate of decline in forced vital capacity in patients treated with nintedanib was similar irrespective of dose adjustments.

**Conclusions:**

The adverse event profile of nintedanib in patients with SSc-ILD is consistent with its established safety and tolerability profile in patients with idiopathic pulmonary fibrosis. Dose adjustment is important to minimise the impact of adverse events and help patients remain on therapy.

**Key messages**

**What is already known about this subject?**

- The adverse event profile of nintedanib in patients with interstitial lung diseases is characterised mainly by gastrointestinal adverse events, particularly diarrhoea, which in most cases are of mild or moderate intensity and do not lead to treatment discontinuation.

**What does this study add?**

- The adverse events associated with nintedanib in patients with systemic sclerosis-associated interstitial lung disease (SSc-ILD) are similar across subgroups defined by age, sex, race and weight.
- Patients with SSc-ILD who have a predisposition to gastrointestinal or intestinal events are not more likely to have gastrointestinal adverse events when treated with nintedanib than patients without such a predisposition.
- The rate of decline in forced vital capacity is similar in nintedanib-treated patients irrespective of dose adjustments used to manage adverse events.

**How might this impact on clinical practice or future developments?**

- The dose adjustments used to manage adverse events in the SENSCIS trial can be implemented in clinical practice to minimise the impact of adverse events and help patients with SSc-ILD remain on nintedanib.

**video abstract:** 

## Introduction

Systemic sclerosis (SSc) is a heterogeneous autoimmune disease characterised by immune dysregulation, microvascular damage, and organ fibrosis.^1^ Interstitial lung disease (ILD) is a common manifestation of SSc and the leading cause of death in patients with SSc.^2^ Nintedanib, an intracellular inhibitor of tyrosine kinases,^3^ has been approved for the treatment of idiopathic pulmonary fibrosis (IPF) and systemic sclerosis-associated interstitial lung disease (SSc-ILD). In the placebo-controlled INPULSIS trials in patients with IPF, SENSCIS trial in patients with SSc-ILD, and INBUILD trial in patients with various fibrosing ILDs with a progressive phenotype, nintedanib reduced the progression of ILD, as demonstrated by a reduction in the rate of decline in forced vital capacity (FVC) over 52 weeks.^4–6^

The adverse event profile of nintedanib in patients with ILDs is characterised mainly by gastrointestinal adverse events.^4–6^ Gastrointestinal issues are also a common problem associated with SSc and with treatments commonly used for SSc.^7^ We used data from the SENSCIS trial to characterise further the safety and tolerability of nintedanib in patients with SSc-ILD and to describe how adverse events were managed during this trial.

## Methods

### Trial design

**Figure 1:**
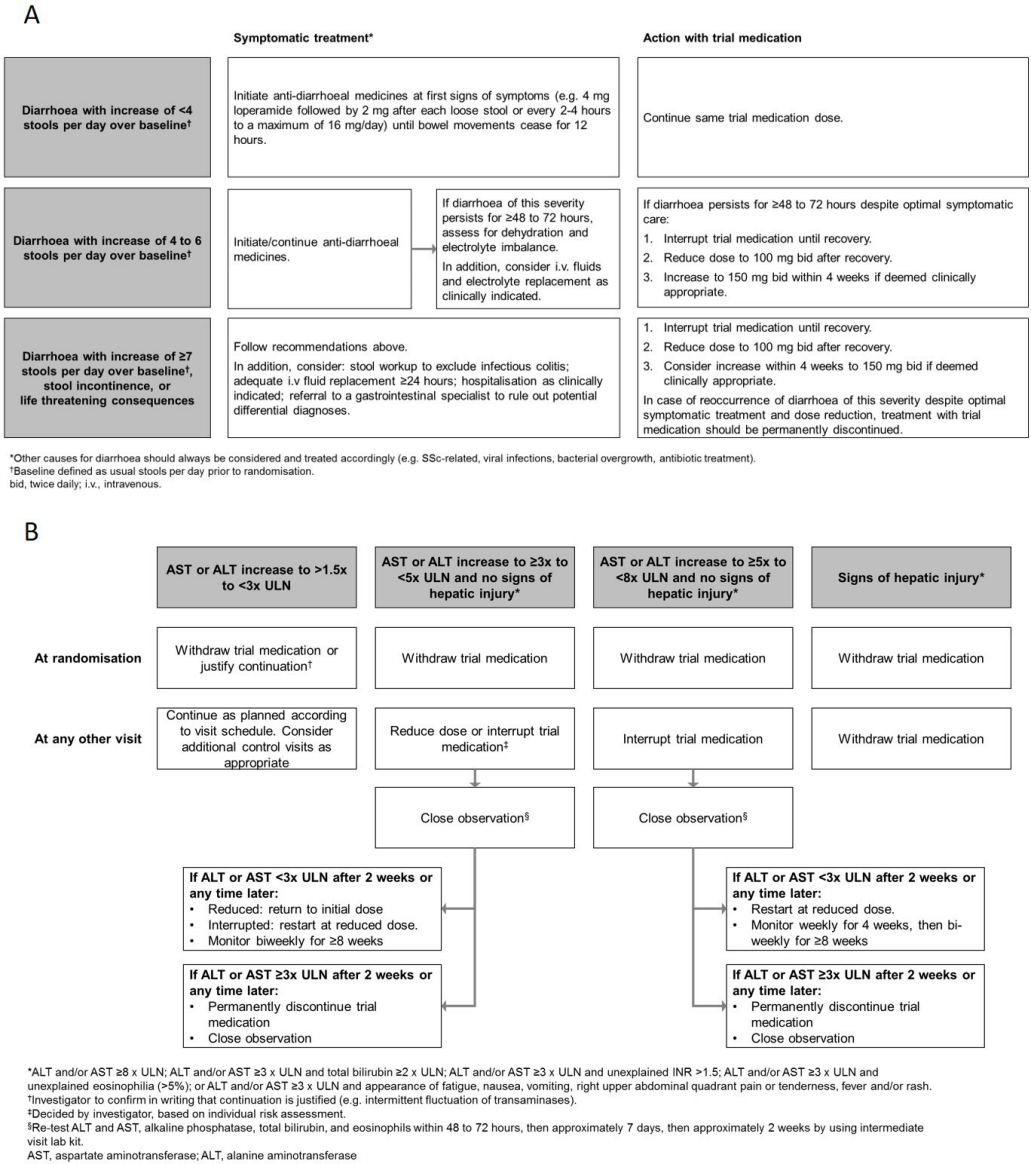
Algorithm for the management of diarrhoea adverse events (A) and hepatic enzyme elevations (B) in the SENSCIS trial. SSc, systemic sclerosis; ULN, upper limit of normal.

The design of the SENSCIS trial has been published, together with the trial protocol.^5^ Briefly, patients with SSc with onset of first non-Raynaud symptom <7 years before screening, extent of fibrotic ILD ≥10% on a high-resolution CT scan, FVC of ≥40% predicted and diffusing capacity of the lung for carbon monoxide 30%–89% predicted were randomised 1:1 to receive nintedanib 150 mg two times per day or placebo. Patients on prednisone ≤10 mg/day (or equivalent) and/or stable therapy with mycophenolate or methotrexate for ≥6 months prior to randomisation were allowed to participate. Patients with investigator-reported significant pulmonary hypertension (PH) (defined as previous clinical or echocardiographic evidence of significant right heart failure, history of right heart catheterisation showing a cardiac index of ≤2 L/min/m² or PH requiring parenteral therapy with epoprostenol/treprostinil) were excluded. Patients without protocol-defined significant PH were allowed to participate. Treatment interruptions (for ≤4 weeks for adverse events considered related to trial medication or ≤8 weeks for other adverse events) and dose reductions to 100 mg two times per day were recommended to manage adverse events. After resolution of the adverse event, nintedanib could be reintroduced or the dose increased back to 150 mg two times per day. Specific recommendations were provided to the investigators for the management of diarrhoea and hepatic enzyme elevations (figure 1). Adverse events were reported by the investigators irrespective of causality and were coded according to the Medical Dictionary for Regulatory Activities V.21.1. Blood samples for pharmacokinetic analyses were collected at weeks 4 and 24, just before drug administration.

### Analyses

Compliance with trial medication was calculated as the number of capsules taken multiplied by 100 and divided by the number of capsules that should have been taken. Dose intensity was defined as the amount of drug administered divided by the amount of drug that would have been received if the dose of 150 mg two times per day had been administered over the 52-week treatment period, or until permanent treatment discontinuation. The annual rate of decline in FVC (mL/year) over 52 weeks (373 days) in patients treated with nintedanib was assessed in subgroups based on dose adjustment, using a random coefficient regression model (with random slopes and intercepts), including effects for antitopoisomerase I antibody status, sex, baseline FVC (mL), age and height and baseline-by-time, treatment-by-subgroup and treatment-by-subgroup-by-time interaction terms.

We analysed adverse events reported over 52 weeks (or until 28 days after the last intake of trial drug in patients who discontinued trial drug before week 52). Based on the mechanism of action of nintedanib^3^ and data from trials in patients with IPF,^4^ we present data on diarrhoea, hepatic adverse events and liver enzyme and bilirubin elevations, bleeding and cardiovascular adverse events. The proportions of patients with any adverse events; with severe, serious and fatal adverse events; with adverse events leading to treatment discontinuation; with hepatic adverse events; and with the most frequent adverse events were assessed in subgroups by age at baseline (<65 years and ≥65 years), sex, weight at baseline (≤65 kg and >65 kg) and race (White, Asian and Black/African–American). Severe adverse events were defined as events that were incapacitating or caused an inability to work or perform usual activities. Serious adverse events were defined as events that resulted in death, were life-threatening, resulted in hospitalisation or prolongation of hospitalisation, resulted in persistent or clinically significant disability or incapacity, were a congenital anomaly or birth defect or were deemed to be serious for any other reason. Gastrointestinal adverse events were analysed in patients with and without a predisposition to intestinal events (defined as a history of and/or presence at baseline of diarrhoea, bloating, constipation and/or incontinence) and in patients with and without a predisposition to gastrointestinal events (defined as a history of gastrointestinal events and/or the presence of gastro-oesophageal reflux disease, oesophageal dysphagia, malabsorption, SSc-related diarrhoea or constipation at baseline). Analyses were descriptive and based on patients who received ≥1 dose of trial drug.

## Results

### Patients

In total, 576 patients were treated. At baseline, the mean (SD) age was 54.0 (12.2) years; weight was 69.7 (15.9) kg; body mass index was 25.9 (5.0) kg/m^2^; 75.2% of patients were female, 67.2% were White and 24.8% were Asian; 60.8% were positive for antitopoisomerase I antibody, and 51.9% had diffuse cutaneous SSc. Most patients (97.7%) were taking ≥1 medication at baseline. Based on customised drug groupings, 79.5% of patients were taking drugs for gastric acid-related disorders; 64.1% were taking antihypertensives; 53.1% were taking therapies for pain; 49.8% were taking corticosteroids; and 30.7% were taking low-dose antithrombotic therapies. Nearly half (48.4%) of patients were taking mycophenolate (mofetil or sodium) and 6.6% were taking methotrexate. The gastrointestinal disorders reported at screening were oesophageal dysphagia or reflux (62.7%), vomiting or early satiety (15.3%), constipation (12.0%), bloating (12.0%), diarrhoea (10.2%) and incontinence (4.7%).

### Dose adjustments and treatment discontinuations

Over 52 weeks, 19.4% of patients in the nintedanib group and 10.8% of patients in the placebo group prematurely discontinued trial medication. Information on dose reductions and treatment interruptions over 52 weeks is shown in table 1. Over 52 weeks, 48.3% of patients in the nintedanib group and 12.2% in the placebo group had ≥1 dose reduction and/or treatment interruption. The most common reason for dose reduction or treatment interruption was diarrhoea. Dose reductions and treatment interruptions were more common among female than male patients (online supplementary table S1).

10.1136/annrheumdis-2020-217331.supp1 Supplementary data

**Table 1:** Dose reductions and treatment interruptions in the SENSCIS trial

	Nintedanib (n=288)	Placebo (n=288)
Patients with ≥1 dose reduction	117 (40.6)	13 (4.5)
Number of dose reductions per patient		
1	104 (36.1)	13 (4.5)
2	13 (4.5)	0
>2	0	0
Time to first dose reduction		
≤30 days	11 (3.8)	2 (0.7)
>30 to ≤61 days	20 (6.9)	4 (1.4)
>61 to ≤91 days	19 (6.6)	1 (0.3)
>91 to ≤182 days	34 (11.8)	4 (1.4)
>182 days	33 (11.5)	2 (0.7)
Patients with ≥1 dose re-escalation following dose reduction	25 (8.7)	2 (0.7)
Patients for whom last dose was 100 mg two times per day	105 (36.5)	11 (3.8)
Most frequent reasons for dose reduction, n (%) of dose reductions*		
Adverse events considered related to trial drug by investigator		
Diarrhoea	77 (59.2)	4 (30.8)
Vomiting	7 (5.4)	0 (0.0)
Alanine aminotransferase increased	5 (3.8)	0 (0.0)
Nausea	5 (3.8)	0 (0.0)
Hepatic enzyme increased	4 (3.1)	1 (7.7)
Abdominal pain upper	3 (2.3)	1 (7.7)
Weight decreased	3 (2.3)	0 (0.0)
Adverse events considered unrelated to trial drug by investigator	4 (3.1)	0 (0.0)
Patients with ≥1 treatment interruption	109 (37.8)	33 (11.5)
Number of treatment interruptions per patient		
1	73 (25.3)	24 (8.3)
2	21 (7.3)	3 (1.0)
>2	15 (5.2)	6 (2.1)
Time to first treatment interruption		
≤30 days	18 (6.3)	8 (2.8)
>30 to ≤61 days	18 (6.3)	6 (2.1)
>61 to ≤91 days	19 (6.6)	6 (2.1)
>91 to ≤182 days	29 (10.1)	3 (1.0)
>182 days	25 (8.7)	10 (3.5)
Duration of treatment interruption (days), mean (SD)^†^	23.1 (17.4)	19.7 (19.8)
Most frequent reasons for treatment interruption, n (%) of interruptions*		
Adverse events considered related to trial drug by investigator		
Diarrhoea	75 (41.2)	12 (19.4)
Abdominal pain upper	19 (10.4)	6 (9.7)
Alanine aminotranferase increased	5 (2.7)	1 (1.6)
Hepatic enzyme increased	5 (2.7)	1 (1.6)
Vomiting	4 (2.2)	1 (1.6)
Hypotension	0 (0.0)	3 (4.8)
Adverse events considered unrelated to trial drug by investigator	19 (10.4)	15 (24.2)

Data are n (%) of patients unless otherwise stated. Dose reductions and treatment interruptions over 52 weeks are shown.

*Reasons reported in >2 patients in either treatment group are shown based on preferred terms in the Medical Dictionary for Regulatory Activities.

†Total duration of all interruptions.

In patients who had a dose reduction after week 4 and were receiving the dose of 100 mg two times per day at week 24 (n=35), the geometric mean trough concentration of nintedanib was 11.50 ng/mL at week 4 and 6.14 ng/mL at week 24. Among patients who did not have a dose reduction between weeks 4 and 24 (n=160), the geometric mean trough concentration of nintedanib was 7.75 ng/mL at week 4 and 7.60 ng/mL at week 24. Compared with patients who did not have a dose reduction, greater proportions of patients who had a dose reduction between weeks 4 and 24 had a baseline weight of ≤65 kg (35.6% vs 54.3%) and were of Asian race (19.4% vs 34.3%).

**Figure 2:**
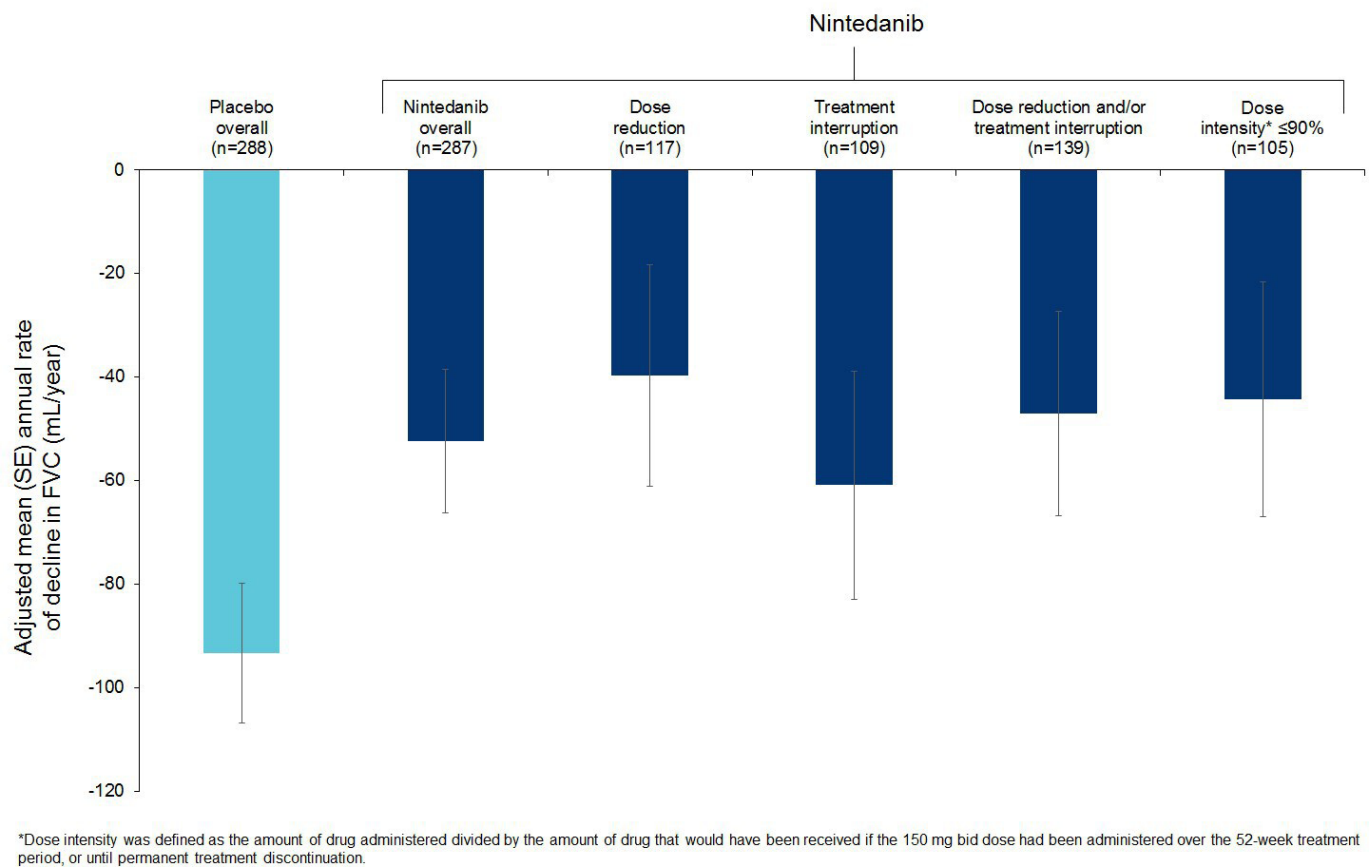
Annual rate of decline in FVC (mL/year) over 52 weeks by dose adjustment in patients treated with nintedanib. FVC, forced vital capacity.

Mean compliance with trial medication was 95.5% in the nintedanib group and 96.4% in the placebo group, and mean dose intensity was 90.3% and 98.4% in these groups, respectively. The median exposure to trial drug (at either dose) over 52 weeks was 12.2 months in both treatment groups. In patients treated with nintedanib, the annual rate of decline in FVC (mL/year) over 52 weeks was similar irrespective of dose adjustments used to manage adverse events (figure 2).

### Adverse events

The proportions of patients with any adverse event/s (98.3% and 95.8%, respectively) and any serious adverse event/s (24.0% and 21.5%, respectively) were similar in the nintedanib and placebo groups. The most common adverse event was diarrhoea, reported in 75.7% of patients in the nintedanib group and 31.6% of patients in the placebo group.

Weight loss was reported as an adverse event by 11.8% of patients in the nintedanib group and 4.5% in the placebo group. At week 52, the mean (SD) change from baseline in weight was −3.22 (4.54) kg in the nintedanib group and −0.25 (4.05) kg in the placebo group.

Adverse events reported in subgroups by sex, age, weight and race are shown in online supplementary tables S2–S5. In the nintedanib group, nausea, vomiting and hepatic events were reported more frequently in female patients, while serious adverse events were more frequent in male patients. Decreased weight and adverse events leading to treatment discontinuation occurred more frequently in nintedanib-treated patients aged ≥65 than<65 years. Liver test abnormalities were reported more frequently in Asian patients than White patients, while nausea and fatigue were more common in White patients than Asian patients. The number of Black/African–American patients was too small to allow conclusions to be drawn about adverse events in this subgroup.

Adverse events leading to permanent discontinuation of trial drug occurred in 16.0% of patients in the nintedanib group and 8.7% of patients in the placebo group. Most of the permanent discontinuations of nintedanib were due to gastrointestinal events, particularly diarrhoea (table 2).

**Table 2:** Adverse events leading to permanent treatment discontinuation in the SENSCIS trial

	Nintedanib (n=288)	Placebo (n=288)
Any adverse event(s) leading to permanent treatment discontinuation	46 (16.0)	25 (8.7)
Most frequent adverse event(s) leading to permanent treatment discontinuation*		
Diarrhoea	20 (6.9)	1 (0.3)
Nausea	6 (2.1)	0
Vomiting	4 (1.4)	1 (0.3)
Abdominal pain upper	3 (1.0)	1 (0.3)
Alanine aminotransferase increased	2 (0.7)	0
Progression of ILD†	3 (1.0)	3 (1.0)

Data are n (%) of patients. Adverse events reported over 52 weeks (or until 28 days after last trial drug intake for patients who discontinued trial drug before week 52).

*Adverse events leading to permanent treatment discontinuation in >2 patients in either treatment group over 52 weeks, coded according to preferred terms in the MedDRA.

†Based on MedDRA preferred term ‘interstitial lung disease’.

ILD, interstitial lung disease; MedDRA, Medical Dictionary for Regulatory Activities.

### Gastrointestinal adverse events

**Figure 3:**
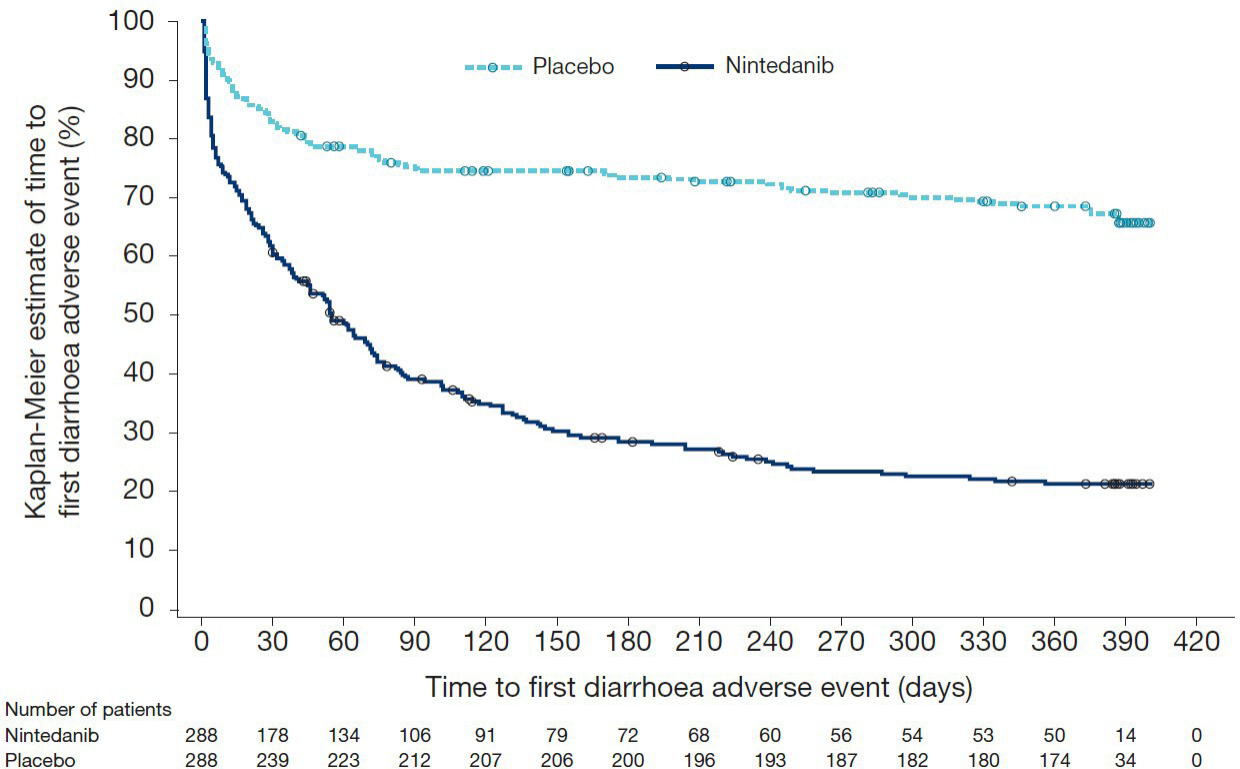
Time to first diarrhoea adverse event in the SENSCIS trial.

Among nintedanib-treated patients who experienced ≥1 diarrhoea adverse event over 52 weeks, approximately half (51.8%) experienced onset of their first event ≤30 days after initiation of treatment (table 3 and figure 3), and most (70.2%) experienced one or two events over 52 weeks. Most (94.5%) experienced events that were at worst of mild or moderate intensity, while 5.5% experienced ≥1 event rated as severe. Most (90.8%) of the nintedanib-treated patients who experienced ≥1 diarrhoea adverse event over 52 weeks did not permanently discontinue the drug due to diarrhoea (table 3). Over 52 weeks, 48% of patients in the nintedanib group and 9% of patients in the placebo group began antidiarrhoeal medication (online supplementary table S6). Additional information on the treatment of diarrhoea adverse events is given in online supplementary table S7.

**Table 3:** Number, intensity and consequences of diarrhoea adverse events in patients who experienced ≥1 diarrhoea adverse event in the SENSCIS trial

	Nintedanib (n=218)	Placebo (n=91)
Number of diarrhoea events		
1	107 (49.1)	54 (59.3)
2	46 (21.1)	21 (23.1)
3	24 (11.0)	4 (4.4)
≥4	41 (18.8)	12 (13.2)
Time to first onset of diarrhoea event		
≤30 days	113 (51.8)	49 (53.8)
>30 to ≤61 days	35 (16.1)	12 (13.2)
>61 to ≤91 days	25 (11.5)	12 (13.2)
>91 to ≤182 days	28 (12.8)	3 (3.3)
>182 days	17 (7.8)	15 (16.5)
Intensity of worst event*		
Mild	108 (49.5)	61 (67.0)
Moderate	98 (45.0)	27 (29.7)
Severe	12 (5.5)	3 (3.3)
Outcome of worst event		
Recovered	202 (92.7)	86 (94.5)
Not yet recovered†	14 (6.4)	5 (5.5)
Recovered/resolved with sequelae	1 (0.5)	0
Unknown	1 (0.5)	0
Consequence of worst event for trial drug		
Permanently discontinued	20 (9.2)	1 (1.1)
Permanent dose reduction	57 (26.1)	2 (2.2)
Neither of above	141 (64.7)	88 (96.7)

Adverse events reported over 52 weeks (or until 28 days after last trial drug intake for patients who discontinued trial drug before week 52). Data are n (% of patients with ≥1 diarrhoea adverse event).

*Mild: awareness of signs or symptoms which are easily tolerated; moderate: enough discomfort to cause interference with usual activity; severe: incapacitating or causing inability to work or to perform the usual activities.

†The patient has not yet returned to his/her previous health status and continues to be followed up.

In both treatment groups, the proportions of patients with adverse events of nausea, vomiting and weight loss were numerically higher in patients with than without a predisposition to intestinal events at baseline (table 4). Other gastrointestinal adverse events in the nintedanib group were reported in similar proportions of patients with and without a predisposition to intestinal events. Similar findings were observed in subgroups with and without a predisposition to gastrointestinal events at baseline (online supplementary table S8).

**Table 4:** Gastrointestinal and weight loss adverse events by predisposition to intestinal events in the SENSCIS trial

	With predisposition to intestinal events		Without predisposition to intestinal events	
	Nintedanib (n=115)	Placebo (n=114)	Nintedanib (n=173)	Placebo (n=174)
Diarrhoea	86 (74.8)	37 (32.5)	132 (76.3)	54 (31.0)
Nausea	38 (33.0)	19 (16.7)	53 (30.6)	20 (11.5)
Vomiting	32 (27.8)	16 (14.0)	39 (22.5)	14 (8.0)
Abdominal pain	13 (11.3)	5 (4.4)	20 (11.6)	16 (9.2)
Weight loss	18 (15.7)	8 (7.0)	16 (9.2)	5 (2.9)

Predisposition to intestinal events was defined as a history of, and/or presence at baseline of, diarrhoea, bloating, constipation and/or incontinence. Data are n (%) of patients with ≥1 such adverse event reported over 52 weeks (or until 28 days after last trial drug intake for patients who discontinued trial drug before week 52). Adverse events shown are those reported in >10% of patients in either the nintedanib or placebo group by single MedDRA preferred terms in the system organ class ‘gastrointestinal disorders’, except for abdominal pain, which was based on a MedDRA high-level term (related preferred terms), and weight loss, which was based on the MedDRA preferred terms ‘weight decreased’ and ‘abnormal loss of weight’.

MedDRA, Medical Dictionary for Regulatory Activities.

### Adverse events in patients with PH

At baseline, 23 patients in the nintedanib group and 29 patients in the placebo group had PH. Among the patients with PH, one patient in each treatment group had an adverse event of PH (indicating worsening of PH) during the trial. There were no events of cardiac failure or pulmonary haemorrhage in patients with PH taking nintedanib. In the nintedanib and placebo groups, respectively, serious adverse events occurred in higher proportions of patients with PH (34.8% and 34.5%) than without PH (23.0% vs 20.1%). In the nintedanib group, the proportions of patients with gastrointestinal adverse events were similar between those with and without PH (online supplementary table S9).

### Other adverse events

The proportions of patients with hepatic adverse events and elevations in hepatic enzymes and bilirubin were greater in patients treated with nintedanib than placebo (table 5). Elevation in alanine aminotransferase (ALT) and/or aspartate aminotransferase (AST) to ≥3× upper limit of normal was reported in 4.9% of patients in the nintedanib group and 0.7% of patients in the placebo group.

**Table 5:** Hepatic adverse events and elevations in hepatic enzymes and bilirubin in the SENSCIS trial

	Nintedanib (n=288)	Placebo (n=288)
**Hepatic adverse event** *	40 (13.9)	9 (3.1)
**Elevation in ALT and/or AST**		
≥3× ULN	14 (4.9)	2 (0.7)
≥5× ULN	3 (1.0)	1 (0.3)
≥8× ULN	0	1 (0.3)
**Elevation in ALT and/or AST≥3× ULN and in bilirubin ≥2× ULN**	0	0
**Elevation in total bilirubin**		
≥1.5× ULN	1 (0.3)	0
≥2× ULN	1 (0.3)	0
**Elevation in alkaline phosphatase**		
≥1.5× ULN	10 (3.5)	3 (1.0)
≥2× ULN	3 (1.0)	0

Data are n (%) of patients with ≥1 such event reported over 52 weeks (or until 28 days after last trial drug intake for patients who discontinued trial drug before week 52).

*Based on the standardised MedDRA query ‘liver related investigations, signs and symptoms’ (broad definition).

ALT, alanine aminotransferase; AST, aspartate aminotransferase; MedDRA, Medical Dictionary for Regulatory Activities; ULN, upper limit of normal.

Bleeding adverse events were reported in 11.1% of patients in the nintedanib group and 8.3% of patients in the placebo group. Epistaxis (2.8% nintedanib and 3.8% placebo) and contusion (2.4% nintedanib and 1.0% placebo) were the most frequently reported bleeding events. Serious bleeding adverse events occurred in 1.4% and 0.7% of patients in the nintedanib and placebo groups, respectively.

Hypertension was reported in 4.9% and 1.7% of patients in the nintedanib and placebo groups, respectively. Other cardiovascular adverse events occurred at a similar low frequency between the treatment groups (online supplementary table S10). Scleroderma renal crisis was reported in one patient (in the nintedanib group).

## Discussion

We used data from the SENSCIS trial to characterise the safety and tolerability profile of nintedanib in patients with SSc-ILD. The adverse event profile of nintedanib in patients with SSc-ILD was consistent with that observed in patients with IPF, with diarrhoea being the most common adverse event.^4 8 9^ Other gastrointestinal adverse events (nausea, vomiting and abdominal pain) and weight loss were also more common in patients treated with nintedanib than placebo.

SSc is commonly associated with gastrointestinal problems such as oesophageal dysmotility, gastro-oesophageal reflux disease, faecal incontinence and small bowel involvement.^7^ In both the nintedanib and placebo groups, gastrointestinal adverse events were more frequently reported in the SENSCIS trial than in the INPULSIS trials in patients with IPF.^8^ However, permanent discontinuations of nintedanib due to diarrhoea occurred in similar proportions of nintedanib-treated patients in SENSCIS as in INPULSIS (6.9% vs 4.4%, respectively). Of note, gastrointestinal adverse events were not more frequent in patients with a predisposition to them, indicating that underlying gastrointestinal disorders should not preclude patients from being treated with nintedanib. The low rate of permanent discontinuation of nintedanib due to gastrointestinal adverse events suggests that the recommendations given to the investigators for the management of adverse events in the SENSCIS trial were effective in managing these events and should be implemented in clinical practice to help maintain patients on treatment.

At baseline, almost half of the patients in the SENSCIS trial were taking mycophenolate, which may be associated with gastrointestinal adverse events.^10^ Previous analyses have shown that the adverse event profile of nintedanib (including the proportion of patients with diarrhoea) was generally similar between subgroups by mycophenolate use, and permanent treatment discontinuations were not more common in nintedanib-treated patients taking mycophenolate, with the caveat that patients taking mycophenolate at baseline had tolerated it for at least 6 months before entering the trial.^11^

The proportion of nintedanib-treated patients who had at least one dose reduction was higher in the SENSCIS trial than in the INPULSIS trials in patients with IPF (40.6% vs 27.9%), while the proportion who prematurely discontinued trial medication over 52 weeks was lower (19.4% vs 24.5%).^8^ As observed in the INPULSIS trials,^12^ the rate of decline in FVC was similar in nintedanib-treated patients irrespective of dose adjustments, suggesting that the dose adjustments used to manage adverse events did not reduce the efficacy of nintedanib in reducing the progression of ILD. However, since these subgroups were defined based on dose adjustments made during treatment, this observation should not be misinterpreted as meaning that patients should be started at a dose of 100 mg two times per day. A dose finding study in patients with IPF identified nintedanib 150 mg two times per day as the optimal dose for reducing the rate of decline in FVC.^13^ While some patients may experience adverse events that require a dose reduction, it is not possible to identify these patients before treatment is initiated and, as such, patients (other than those with mild hepatic impairment^14^) should initiate treatment at a dose of 150 mg two times per day to ensure that they receive the optimal drug effect. In patients with IPF, a positive relationship was observed between exposure to nintedanib and elevations in liver enzymes (ALT and/or AST≥3× ULN). Females may have a higher exposure-adjusted risk of ALT and/or AST≥3× ULN, but with a low overall occurrence.^14^ Higher age, lower weight and Asian race were associated with small to moderate increases in nintedanib exposure, which were within the range of interpatient variability and did not warrant a priori dose adjustment.^15 16^

Nintedanib is an inhibitor of the vascular endothelial growth factor receptor (VEGFR)^3^ and VEGFR inhibition is associated with an increased risk of bleeding.^17^ Patients at high risk of bleeding have been excluded from clinical trials of nintedanib, although use of low-dose antithrombotics and analgesics was permitted in the SENSCIS trial. In the SENSCIS trial, bleeding adverse events were reported in 11.1% of patients in the nintedanib group compared with 8.3% in the placebo group. Vascular endothelial growth factor promotes wound healing through several mechanisms, including collagen deposition, angiogenesis and epithelialisation.^18^ Thus, it is notable that there was no difference between nintedanib and placebo in net digital ulcer burden.^5^ No indication of aggravation of PH, cardiac failure or pulmonary haemorrhage was observed in patients with PH treated with nintedanib.

As observed in previous studies,^9^ hepatic adverse events and liver test abnormalities were more common in patients treated with nintedanib than placebo in the SENSCIS trial. Most of the observed elevations in ALT and/or AST were <3 times the upper limit of normal. It is recommended that liver function tests be conducted prior to initiation of treatment with nintedanib, at regular intervals during the first 3 months of treatment and periodically thereafter or as clinically indicated.^14^ No patient met criteria for Hy’s law.

Although long-term data on the use of nintedanib in patients with SSc-ILD are not yet available, data from the open-label extension of the INPULSIS trials, INPULSIS-ON, suggest that the safety and tolerability of nintedanib are maintained with long-term use.^19^ In addition, pharmacovigilance data collected in patients with IPF, based on over 80 000 patient-years of exposure, suggest that the safety and tolerability of nintedanib in clinical practice is consistent with that observed in clinical trials.^20^

Limitations of these analyses include incomplete information on the procedures used by the investigators used to manage gastrointestinal adverse events and on adverse events reported in patients taking specific comedications (other than mycophenolate). When comparing results between the INPULSIS and SENSCIS trials, differences in the patient populations need to be kept in mind, particularly that IPF is a non-systemic disease that tends to present in male ex-smokers in the sixth or seventh decade of life, while SSc is a systemic multiorgan autoimmune disease that is more common in women, with a peak onset in middle age, and that the patients in the SENSCIS trial had a lower mean body mass index.

In conclusion, these data from the SENSCIS trial demonstrate that the adverse event profile of nintedanib in patients with SSc-ILD is consistent with its established safety and tolerability profile in patients with IPF. Adverse events associated with nintedanib were generally consistent across subgroups defined by age, sex, race and weight, although nausea, vomiting and hepatic adverse events were reported more frequently in female than male patients. The effect of nintedanib on reducing the rate of decline in FVC was similar irrespective of dose adjustments used to manage adverse events. Management of adverse events using symptomatic therapies and dose adjustment is important to minimise the impact of adverse events and to help patients remain on therapy.
