# Supplementary material for: Safety and tolerability of nintedanib in patients with systemic sclerosis-associated interstitial lung disease: data from the SENSCIS trial
Source: Ann Rheum Dis. 2020 Aug 5;79(11):1478–84. doi: 10.1136/annrheumdis-2020-217331 (PMC7569371; doi:10.1136/annrheumdis-2020-217331)
Supplement: Supplementary data [file annrheumdis-2020-217331supp001.pdf]

Online supplementary files

Table S1. Dose reductions and treatment interruptions in the SENSCIS trial in subgroups by sex

	Female		Male	
	**Nintedanib (n=221)**	**Placebo (n=212)**	**Nintedanib (n=67)**	**Placebo (n=76)**
Patients with ≥1 dose reduction	105 (47.5)	11 (5.2)	12 (17.9)	2 (2.6)
Number of dose reductions per patient				
1	95 (43.0)	11 (5.2)	9 (13.4)	2 (2.6)
2	10 (4.5)	0	3 (4.5)	0
>2	0	0	0	0
Time to first dose reduction				
≤30 days	11 (5.0)	2 (0.9)	0	0
>30 to ≤61 days	19 (8.6)	3 (1.4)	1 (1.5)	1 (1.3)
>61 to ≤91 days	17 (7.7)	1 (0.5)	2 (3.0)	0
>91 to ≤182 days	29 (13.1)	4 (1.9)	5 (7.5)	0
>182 days	29 (13.1)	1 (0.5)	4 (6.0)	1 (1.3)
Patients with ≥1 dose re-escalation following dose reduction	22 (10.0)	2 (0.9)	3 (4.5)	0
Patients for whom last dose was 100 mg bid	93 (42.1)	9 (4.2)	12 (17.9)	2 (2.6)
Patients with ≥1 treatment interruption	92 (41.6)	25 (11.8)	17 (25.4)	8 (10.5)
Number of treatment interruptions per patient				
1	61 (27.6)	16 (7.5)	12 (17.9)	8 (10.5)
2	18 (8.1)	3 (1.4)	3 (4.5)	0
>2	13 (5.9)	6 (2.8)	2 (3.0)	0
Time to first treatment				

interruption				
≤30 days	17 (7.7)	8 (3.8)	1 (1.5)	0
>30 to ≤61 days	16 (7.2)	5 (2.4)	2 (3.0)	1 (1.3)
>61 to ≤91 days	17 (7.7)	4 (1.9)	2 (3.0)	2 (2.6)
>91 to ≤182 days	23 (10.4)	1 (0.5)	6 (9.0)	2 (2.6)
>182 days	19 (8.6)	7 (3.3)	6 (9.0	3 (3.9)
Duration of treatment interruption/s(days), mean (SD)*	23.7 (17.8)	21.7 (22.0)	20.0 (15.0)	13.3 (8.3)

Data are n (%) of patients unless otherwise stated. Dose reductions and treatment interruptions over 52 weeks are shown. *Total duration of all interruptions.

Table S2. Adverse events in the SENSCIS trial in subgroups by sex

	Female		Male	
	Nintedanib (n=221)	Placebo (n=212)	Nintedanib (n=67)	Placebo (n=76)
Any adverse event(s)	219 (99.1)	204 (96.2)	64 (95.5)	72 (94.7)
Most frequent adverse events*				
Diarrhoea	165 (74.7)	66 (31.1)	53 (79.1)	25 (32.9)
Nausea	78 (35.3)	29 (13.7)	13 (19.4)	10 (13.2)
Vomiting	62 (28.1)	19 (9.0)	9 (13.4)	11 (14.5)
Skin ulcer	42 (19.0)	37 (17.5)	11 (16.4)	13 (17.1)
Nasopharyngitis	27 (12.2)	36 (17.0)	9 (13.4)	13 (17.1)
Cough	23 (10.4)	37 (17.5)	11 (16.4)	15 (19.7)
Upper respiratory tract infection	26 (11.8)	27 (12.7)	7 (10.4)	8 (10.5)
Abdominal pain	26 (11.8)	17 (8.0)	7 (10.4)	4 (5.3)
Fatigue	23 (10.4)	13 (6.1)	8 (11.9)	7 (9.2)
Weight decreased	27 (12.2)	7 (3.3)	7 (10.4)	5 (6.6)
Hepatic events[†]	34 (15.4)	6 (2.8)	6 (9.0)	3 (3.9)
Adverse event(s) leading to treatment discontinuation	37 (16.7)	18 (8.5)	9 (13.4)	7 (9.2)
Severe adverse event(s)[‡]	38 (17.2)	22 (10.4)	14 (20.9)	14 (18.4)
Serious adverse event(s)[§]	47 (21.3)	43 (20.3)	22 (32.8)	19 (25.0)
Fatal adverse event	2 (0.9)	2 (0.9)	3 (4.5)	2 (2.6)

Data shown are n (%) of patients with ≥1 such event reported over 52 weeks (or until 28 days after last trial drug intake for patients who discontinued trial drug before week 52. *Events reported in >10% of

patients in either treatment group in the overall population, coded based on MedDRA preferred terms. †Based on the standardised MedDRA query “liver related investigations, signs and symptoms” (broad definition). ‡Adverse events that were incapacitating or caused an inability to work or perform usual activities. §Adverse events that resulted in death, were life-threatening, resulted in hospitalisation or prolongation of hospitalisation, resulted in persistent or clinically significant disability or incapacity, were a congenital anomaly or birth defect, or were deemed to be serious for any other reason. MedDRA, Medical Dictionary for Regulatory Activities.

Table S3. Adverse events in the SENSCIS trial in subgroups by age at baseline

	Age <65 years		Age ≥65 years	
	Nintedanib (n=224)	Placebo (n=229)	Nintedanib (n=64)	Placebo (n=59)
Any adverse event(s)	219 (97.8)	221 (96.5)	64 (100.0)	55 (93.2)
Most frequent adverse events*				
Diarrhoea	167 (74.6)	74 (32.3)	51 (79.7)	17 (28.8)
Nausea	71 (31.7)	34 (14.8)	20 (31.3)	5 (8.5)
Vomiting	53 (23.7)	24 (10.5)	18 (28.1)	6 (10.2)
Skin ulcer	42 (18.8)	45 (19.7)	11 (17.2)	5 (8.5)
Nasopharyngitis	28 (12.5)	39 (17.0)	8 (12.5)	10 (16.9)
Cough	26 (11.6)	42 (18.3)	8 (12.5)	10 (16.9)
Upper respiratory tract infection	28 (12.5)	32 (14.0)	5 (7.8)	3 (5.1)
Abdominal pain	24 (10.7)	19 (8.3)	9 (14.1)	2 (3.4)
Fatigue	23 (10.3)	19 (8.3)	8 (12.5)	1 (1.7)
Weight decreased	22 (9.8)	9 (3.9)	12 (18.8)	3 (5.1)
Liver test abnormalities[†]	29 (12.9)	8 (3.5)	11 (17.2)	1 (1.7)
Adverse event(s) leading to treatment discontinuation	33 (14.7)	16 (7.0)	13 (20.3)	9 (15.3)
Severe adverse event(s)[‡]	42 (18.8)	27 (11.8)	10 (15.6)	9 (15.3)
Serious adverse event(s)[§]	53 (23.7)	47 (20.5)	16 (25.0)	15 (25.4)
Fatal adverse event	4 (1.8)	2 (0.9)	1 (1.6)	2 (3.4)

Data shown are n (%) of patients with ≥1 such event reported over 52 weeks (or until 28 days after last trial drug intake for patients who discontinued trial drug before week 52. *Events reported in >10% of

patients in either treatment group in the overall population, coded based on MedDRA preferred terms. †Based on the standardised MedDRA query "liver related investigations, signs and symptoms" (broad definition). ‡Adverse events that were incapacitating or caused an inability to work or perform usual activities. §Adverse events that resulted in death, were life-threatening, resulted in hospitalisation or prolongation of hospitalisation, resulted in persistent or clinically significant disability or incapacity, were a congenital anomaly or birth defect, or were deemed to be serious for any other reason. MedDRA, Medical Dictionary for Regulatory Activities.

Table S4. Adverse events in the SENSCIS trial in subgroups by weight at baseline

	≤65 kg		>65 kg	
	**Nintedanib (n=120)**	**Placebo (n=117)**	**Nintedanib (n=168)**	**Placebo (n=171)**
Any adverse event(s)	119 (99.2)	111 (94.9)	164 (97.6)	165 (96.5)
Most frequent adverse events*				
Diarrhoea	86 (71.7)	29 (24.8)	132 (78.6)	62 (36.3)
Nausea	33 (27.5)	16 (13.7)	58 (34.5)	23 (13.5)
Vomiting	26 (21.7)	12 (10.3)	45 (26.8)	18 (10.5)
Skin ulcer	31 (25.8)	23 (19.7)	22 (13.1)	27 (15.8)
Nasopharyngitis	13 (10.8)	24 (20.5)	23 (13.7)	25 (14.6)
Cough	8 (6.7)	22 (18.8)	26 (15.5)	30 (17.5)
Upper respiratory tract infection	12 (10.0)	13 (11.1)	21 (12.5)	22 (12.9)
Abdominal pain	15 (12.5)	9 (7.7)	18 (10.7)	12 (7.0)
Fatigue	8 (6.7)	6 (5.1)	23 (13.7)	14 (8.2)
Weight decreased	15 (12.5)	5 (4.3)	19 (11.3)	7 (4.1)
Liver test abnormalities†	16 (13.3)	6 (5.1)	24 (14.3)	3 (1.8)
Adverse event(s) leading to treatment discontinuation	25 (20.8)	12 (10.3)	21 (12.5)	13 (7.6)
Severe adverse event(s)‡	22 (18.3)	14 (12.0)	30 (17.9)	22 (12.9)
Serious adverse event(s)§	29 (24.2)	26 (22.2)	40 (23.8)	36 (21.1)
Fatal adverse event	2 (1.7)	2 (1.7)	3 (1.8)	2 (1.2)

Data shown are n (%) of patients with ≥1 such event reported over 52 weeks (or until 28 days after last trial drug intake for patients who discontinued trial drug before week 52. *Events reported in >10% of

patients in either treatment group in the overall population, coded based on MedDRA preferred terms. [†]Based on the standardised MedDRA query "liver related investigations, signs and symptoms" (broad definition). [‡]Adverse events that were incapacitating or caused an inability to work or perform usual activities. [§]Adverse events that resulted in death, were life-threatening, resulted in hospitalisation or prolongation of hospitalisation, resulted in persistent or clinically significant disability or incapacity, were a congenital anomaly or birth defect, or were deemed to be serious for any other reason. MedDRA, Medical Dictionary for Regulatory Activities.

Table S5. Adverse events in the SENSCIS trial in subgroups by race

	**White**		**Asian**		**Black/African-American**	
	**Nintedanib (n=201)**	**Placebo (n=186)**	**Nintedanib (n=62)**	**Placebo (n=81)**	**Nintedanib (n=20)**	**Placebo (n=16)**
Any adverse event(s)	196 (97.5)	180 (96.8)	62 (100.0)	77 (95.1)	20 (100.0)	14 (87.5)
Most frequent adverse events*						
Diarrhoea	146 (72.6)	62 (33.3)	50 (80.6)	23 (28.4)	17 (85.0)	3 (18.8)
Nausea	73 (36.3)	31 (16.7)	13 (21.0)	6 (7.4)	5 (25.0)	0
Vomiting	52 (25.9)	25 (13.4)	13 (21.0)	3 (3.7)	6 (30.0)	0
Skin ulcer	33 (16.4)	35 (18.8)	15 (24.2)	11 (13.6)	5 (25.0)	2 (12.5)
Nasopharyngitis	26 (12.9)	31 (16.7)	10 (16.1)	16 (19.8)	0	0
Cough	26 (12.9)	40 (21.5)	4 (6.5)	9 (11.1)	4 (20.0)	2 (12.5)
Weight decreased	25 (12.4)	9 (4.8)	8 (12.9)	2 (2.5)	1 (5.0)	1 (6.3)
Upper respiratory tract infection	21 (10.4)	21 (11.3)	10 (16.1)	11 (13.6)	1 (5.0)	2 (12.5)
Abdominal pain	26 (12.9)	15 (8.1)	5 (8.1)	5 (6.2)	2 (10.0)	1 (6.3)
Fatigue	25 (12.4)	16 (8.6)	2 (3.2)	2 (2.5)	4 (20.0)	1 (6.3)
Liver test abnormalities†	25 (12.4)	6 (3.2)	12 (19.4)	3 (3.7)	3 (15.0)	0
Adverse event(s) leading to treatment discontinuation	33 (16.4)	17 (9.1)	9 (14.5)	5 (6.2)	3 (15.0)	3 (18.8)
Severe adverse event(s)‡	33 (16.4)	25 (13.4)	10 (16.1)	6 (7.4)	8 (40.0)	5 (31.3)

Serious adverse event(s)[§]	45 (22.4)	43 (23.1)	16 (25.8)	12 (14.8)	8 (40.0)	7 (43.8)
Fatal adverse event	1 (0.5)	3 (1.6)	1 (1.6)	1 (1.2)	3 (15.0)	0

Data shown are n (%) of patients with ≥1 such event reported over 52 weeks (or until 28 days after last trial drug intake for patients who discontinued trial drug before week 52. *Events reported in >10% of patients in either treatment group in the overall population, coded based on MedDRA preferred terms. [†]Based on the standardised MedDRA query “liver related investigations, signs and symptoms” (broad definition). [‡]Adverse events that were incapacitating or caused an inability to work or perform usual activities. [§]Adverse events that resulted in death, were life-threatening, resulted in hospitalisation or prolongation of hospitalisation, resulted in persistent or clinically significant disability or incapacity, were a congenital anomaly or birth defect, or were deemed to be serious for any other reason. MedDRA, Medical Dictionary for Regulatory Activities.

Table S6. Anti-diarrhoeal therapies taken at baseline or initiated over 52 weeks of treatment

	**Nintedanib (n=288)**	**Placebo (n=288)**
**At baseline**		
Total	12 (4.2)	8 (2.8)
Loperamide hydrochloride	12 (4.2)	5 (1.7)
Loperamide	0	2 (0.7)
Houttuynia cordata herb	0	1 (0.3)
**Initiated over 52 weeks***		
Total	138 (47.9)	26 (9.0)
Loperamide hydrochloride	86 (29.9)	15 (5.2)
Loperamide	56 (19.4)	9 (3.1)
Diphenoxylate/atropine	1 (0.3)	2 (0.7)
Menthol	0	2 (0.7)

Based on customised drug grouping “antidiarrheal”. Data are n (%) of patients. *Initiated between first drug intake and week 52 or last trial drug intake, whichever was earlier.

Table S7. Treatment of diarrhoea adverse events (based on information provided in specific pages of case report form)

	Nintedanib (n=288)	Placebo (n=288)
Number of patients for whom ≥1 page* was completed	220	93
Number of pages* completed	613	196
Diarrhoea event responded to treatment, n (%)	264 (43.1)	51 (26.0)
Diarrhoea event did not respond to treatment, n (%)	107 (17.5)	22 (11.2)
Diarrhoea event was not treated, n (%)	215 (35.1)	119 (60.7)
Missing treatment information, n (%)	27 (4.4)	4 (2.0)

*Investigators were asked to complete a specific page of the case report form for every event of diarrhoea.

Table S8. Gastrointestinal and weight loss adverse events by predisposition to gastrointestinal events in the SENSCIS trial

	**With predisposition to gastrointestinal events**		**Without predisposition to gastrointestinal events**	
	**Nintedanib (n=234)**	**Placebo (n=235)**	**Nintedanib (n=54)**	**Placebo (n=53)**
Diarrhoea	180 (76.9)	80 (34.0)	38 (70.4)	11 (20.8)
Nausea	74 (31.6)	35 (14.9)	17 (31.5)	4 (7.5)
Vomiting	60 (25.6)	28 (11.9)	11 (20.4)	2 (3.8)
Abdominal pain	44 (18.8)	29 (12.3)	9 (16.7)	3 (5.7)
Weight loss	31 (13.2)	12 (5.1)	3 (5.6)	1 (1.9)

Predisposition to gastrointestinal events was defined as a history of gastrointestinal events, and/or the presence of gastroesophageal reflux disease, oesophageal dysphagia, malabsorption, or SSc-related diarrhea or constipation at baseline. Data are n (%) of patients with ≥1 such adverse event reported over 52 weeks (or until 28 days after last trial drug intake for patients who discontinued trial drug before week 52). Adverse events shown are those reported in >10% of patients in either the nintedanib or placebo group by single MedDRA preferred terms in the system organ class “gastrointestinal disorders”, except for abdominal pain, which was based on a MedDRA high-level term (related preferred terms), and weight loss, which was based on the MedDRA preferred terms “weight decreased” and “abnormal loss of weight”. MedDRA, Medical Dictionary for Regulatory Activities; SSc, systemic sclerosis.

Table S9. Adverse events by presence of investigator-reported significant pulmonary hypertension at baseline in the SENSCIS trial

	With pulmonary hypertension at baseline*		Without pulmonary hypertension at baseline*	
	**Nintedanib (n=23)**	**Placebo (n=29)**	**Nintedanib (n=265)**	**Placebo (n=259)**
Any adverse event(s)	23 (100.0)	29 (100.0)	260 (98.1)	247 (95.4)
Gastrointestinal adverse events*				
Diarrhoea	18 (78.3)	13 (44.8)	200 (75.5)	78 (30.1)
Nausea	9 (39.1)	5 (17.2)	82 (30.9)	34 (13.1)
Vomiting	6 (26.1)	5 (17.2)	65 (24.5)	25 (9.7)
Abdominal pain	4 (17.4)	4 (13.8)	49 (18.5)	28 (10.8)
Weight loss†	2 (8.7)	0 (0.0)	32 (12.1)	13 (5.0)
Respiratory conditions‡	13 (56.5)	14 (48.3)	88 (33.2)	97 (37.5)
Serious adverse event(s)§	8 (34.8)	10 (34.5)	61 (23.0)	52 (20.1)

*Patients with investigator-reported significant pulmonary hypertension (defined as previous clinical or echocardiographic evidence of significant right heart failure, history of right heart catheterisation showing a cardiac index ≤2 L/min/m², or pulmonary hypertension requiring parenteral therapy with epoprostenol/treprostinil) were excluded from the trial. Data are n (%) of patients with ≥1 such adverse event reported over 52 weeks (or until 28 days after last trial drug intake for patients who discontinued trial drug before week 52). *Adverse events shown are those reported in >10% of patients in the nintedanib or placebo group by single MedDRA preferred terms in the system organ class "gastrointestinal disorders", except for abdominal pain, which was based on a MedDRA high-level term (related preferred terms). †Based on the MedDRA preferred terms "weight decrease" and "abnormal loss of weight". ‡System organ class "respiratory, thoracic and mediastinal disorders". §Adverse events that resulted in death, were life-threatening, resulted in hospitalisation or prolongation of hospitalisation, resulted in persistent or clinically significant disability or incapacity, were a congenital anomaly or birth

defect, or were deemed to be serious for any other reason. MedDRA, Medical Dictionary for Regulatory Activities.

Table S10. Cardiovascular adverse events in the SENSCIS trial

	**Nintedanib (n=288)**	**Placebo (n=288)**
Hypertension*	14 (4.9)	5 (1.7)
Major adverse cardiovascular events†	4 (1.4)	5 (1.7)
Venous thromboembolism*	4 (1.4)	3 (1.0)
Haemorrhagic and ischaemic stroke*	3 (1.0)	1 (0.3)
Arterial thromboembolism*	2 (0.7)	2 (0.7)
QT prolongation*	2 (0.7)	0
Myocardial infarction*	0	2 (0.7)
Cardiac failure*	1 (0.3)	1 (0.3)
Deep vein thrombosis‡	1 (0.3)	0
Pulmonary embolism‡	0	1 (0.3)

Data are n (%) of patients with ≥1 such adverse event reported over 52 weeks (or until 28 days after last trial drug intake for patients who discontinued trial drug before week 52). *Narrow standardised MedDRA query. †Based on fatal adverse events in the MedDRA system organ classes “cardiac disorders” and “vascular disorders”; any fatal and non-fatal events in the subordinate standardised MedDRA query “myocardial infarction” (broad); any fatal and non-fatal stroke events (based on selected MedDRA preferred terms); and the MedDRA preferred terms “sudden death”, “cardiac death” and “sudden cardiac death”. ‡Single MedDRA preferred term. MedDRA, Medical Dictionary for Regulatory Activities.

Data sharing statement

To ensure independent interpretation of clinical study results, Boehringer Ingelheim grants all external authors access to all relevant material, including participant-level clinical study data, and relevant material as needed by them to fulfill their role and obligations as authors under the ICMJE criteria. Furthermore, clinical study documents (e.g. study report, study protocol, statistical analysis plan) and participant clinical study data are available to be shared after publication of the primary manuscript in a peer-reviewed journal and if regulatory activities are complete and other criteria met per the BI Policy on Transparency and Publication of Clinical Study Data: https://trials.boehringer-ingelheim.com/

Prior to providing access, documents will be examined, and, if necessary, redacted and the data will be de-identified, to protect the personal data of study participants and personnel, and to respect the boundaries of the informed consent of the study participants.

Clinical Study Reports and Related Clinical Documents can also be requested via the link: https://trials.boehringer-ingelheim.com/

All requests will be governed by a Document Sharing Agreement.

Bona fide, qualified scientific and medical researchers may request access to de-identified, analysable participant clinical study data with corresponding documentation describing the structure and content of the datasets. Upon approval, and governed by a Data Sharing Agreement, data are shared in a secured data-access system for a limited period of 1 year, which may be extended upon request.

Researchers should use the https://trials.boehringer-ingelheim.com/ link to request access to study data.
